# The effect of creatine on the *ex vivo* contractility of myometrial tissue derived from term pregnant mice and women

**DOI:** 10.1080/15502783.2025.2533675

**Published:** 2025-07-18

**Authors:** Alexus J. Brown, Jeff Reese, Jennifer L. Herington, Stacey J. Ellery

**Affiliations:** a Vanderbilt University Medical Center, Division of Neonatology, Department of Pediatrics, Nashville, TN, USA; b Vanderbilt University, Department of Pharmacology, Nashville, TN, USA; c The Ritchie Centre, Hudson Institute of Medical Research, Clayton, VIC, Australia; d Monash University, Department of Obstetrics and Gynaecology, Clayton, VIC, Australia

**Keywords:** Creatine, myometrium, muscle contraction, oxytocin, pregnancy, organ culture techniques

## Abstract

**Background:**

There is evidence that the smooth muscle layer of the uterus, the myometrium, uses creatine to support energy turnover in pregnancy and labour. Our study examined the effects of creatine supplementation on *ex vivo* myometrial contractility, hypothesising that it enhances spontaneous contractility and reduces fatigue in spontaneous and oxytocin-induced contractions.

**Methods:**

Human myometrial tissue was collected from consenting patients at the time of cesarean deliveries at term gestation (> 37 weeks). Strips (1cm x 0.5cm x 1cm) were incubated overnight in oxygenated Krebs bicarbonate buffer (KBB) ± 5mM creatine and placed under 3g of tension in organ baths (37°C, 95%O2, 5%CO2) containing fresh KBB ± creatine and allowed to develop spontaneous contractions. Murine myometrial strips were obtained at term gestation (day 19), placed under 1g tension in organ baths, and allowed to establish rhythmic contractions. Strips were treated with 5mM of creatine for 3 hours or cumulative additive concentrations (0.05, 0.1, 0.5, 1, and 5mM) every 30 minutes. After 3 hours of spontaneous contractions, human and murine samples received 1nM of oxytocin and contracted for an additional 2 hours. Contractile activity was recorded with amplitude, frequency, and area under the curve (AUC) analyzed. All time-course (1, 2, 3 hrs) and oxytocin treatment data were expressed as a percent change from baseline spontaneous contractile activity at 40 minutes and compared using a 2-way ANOVA.

**Results:**

There were no statistically significant differences between spontaneous contractility over time (-51% vs -51%, human; 5% vs -25%, mouse) in myometrium supplemented with creatine or vehicle, respectively. Furthermore, there were no statistically significant differences in oxytocin-induced contractility at 0hr (108% vs 156%, human; 83% vs 110%, mouse) or 2hrs (-16% vs -21%, human) -19% vs -21%, mouse) between myometrium supplemented with creatine or vehicle, respectively.

**Conclusions:**

Creatine treatment did not affect human or murine *ex vivo* myometrial contractility.

